# Barriers and enablers of TB infection screening and treatment programme for recent migrants in East London

**DOI:** 10.5588/pha.25.0041

**Published:** 2026-03-06

**Authors:** K. O’Brien, S. Ikram, M. Burman, A. Rahman, P. Patel, S. Dart, D. Trathen, D. Zenner, A.M. Malhotra, H. Kunst

**Affiliations:** 1 Barts and The London School of Medicine and Dentistry, Queen Mary University of London, London, UK;; 2 NHS North East London ICB, London, UK;; 3 Homerton Healthcare NHS Foundation Trust, Respiratory Medicine, London, UK;; 4 Queen Mary University of London Faculty of Medicine and Dentistry, Blizard Institute, London, UK;; 5 Barking, Havering and Redbridge NHS Trust, Romford, UK;; 6 Community Pharmacy, Community Pharmacy England, North East London, UK;; 7 Barts Health NHS Trust, Respiratory Medicine, London, UK;; 8 Queen Mary University of London Wolfson Institute of Population Health, Global Public Health Unit, London, UK;; 9 Queen Mary and Barts Health Tuberculosis Centre, Faculty of Medicine and Dentistry, Queen Mary University of London, London, UK;; 10 Clinical Sciences Department, Liverpool School of Tropical Medicine, Liverpool, UK.

**Keywords:** tuberculosis, migrant health, service delivery

## Abstract

**BACKGROUND:**

The majority of active TB cases in low-burden, high-income settings arise from reactivation of TB infection (TBI). The London Borough of Newham, UK, piloted a novel screening and treatment TBI programme for recent migrants. This was situated entirely within primary care.

**OBJECTIVE AND DESIGN:**

This study aims to highlight key enablers and barriers to delivering a TBI programme in primary care. Views of health care professionals and relevant stakeholders were sought through questionnaires and semi-structured interviews.

**RESULTS:**

Perspectives from 43 health care professionals are included. A perceived ‘good relationship’ between patients and health care professionals was the most commonly cited enablers across groups, followed by education and training of service providers. Physicians reported time constraints as a common barrier, whereas pharmacists were more likely to identify low levels of patient knowledge surrounding TBI as a barrier to engagement. Enablers identified by stakeholders included effective communication between stakeholders and training of service providers. Aggregate data collection and monitoring was considered a significant enabler, as was patient education by health care professionals and novel educational tools.

**CONCLUSION:**

Community-based TBI programmes can be successful. Key enablers include TBI-specific training with communities and amongst health care professionals, collaboration between health care professionals and stakeholders, and aggregate data monitoring.

One quarter of the world’s population is estimated to be infected with *Mycobacterium tuberculosis* (*M.TB*).^1,2^ Amongst individuals infected with *M.TB*, it is estimated that 5%–10% will develop active TB disease during their lifetime, although the mechanism behind this is largely unknown.^3^ In many high-income settings, including England, TB incidence has gradually declined over recent decades. The majority of active TB cases arise from reactivation of TB infection (TBI), particularly amongst at-risk groups including migrants.^1,4^ A key component of England’s TB action plan is to improve screening and treatment of TBI amongst recent migrants.^5,6^ Identification and treatment of TBI is a key component of the WHO’s strategy to eliminate TB.^7^ In the UK, the national TBI screening programme targets migrants aged 16–35 years from countries with a TB incidence of ≥150 cases per 100,000 population, or from any country in sub-Saharan Africa, within the past 5 years. Eligible individuals are offered TBI testing via an interferon-gamma release assay (IGRA), typically following a normal chest radiograph, which is used to exclude active TB disease before TBI treatment is initiated. Many factors are known to affect treatment completion rates including the choice of the TBI treatment and therefore shorter rifampicin-based regimens such as 3 months of isoniazid and rifampicin (3HR) are used.^8^

Traditionally, TBI screening and treatment in the UK occurs in hospital-based TB clinics (secondary care). Existing models of care have been associated with low rates of treatment completion, particularly amongst migrant communities.^9^ Reasons are likely multifactorial, including asymptomatic presentation, an individual’s perception of low risk of developing TB, health-related stigma and fear of discrimination, potential side effects of treatment and the amount of time required to engage with health care facilities.^10,11^ Further, secondary care services are associated with higher delivery costs and are criticised for taking away acute care capacity, for example, active TB cases.^12^ In 2014, the London Borough of Newham became a pilot site for the national TBI screening programme for recent migrants. Newham has a high migrant population and the highest incidence of active TB within the UK.^13^ The local body within the National Health Service (NHS) responsible for planning and funding health care services for their local population – the Newham Clinical Commissioning Group (CCG) – developed a novel model in which TBI screening and treatment was offered entirely within primary care (Figure 1).^13^

**FIGURE 1.**
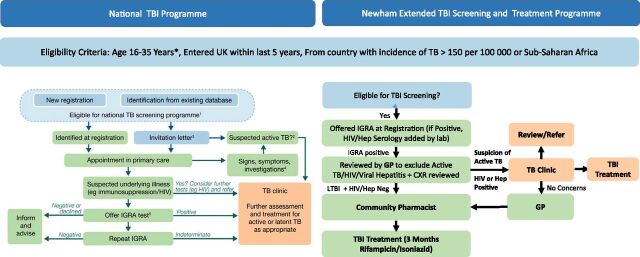
Comparing National Model of Care for Tuberculosis Screening (TBI) in England (left) and the Local Model of Care used in the London Borough of Newham (right). TB preventive therapy (TPT) is provided by general practitioners, whilst pharmacists oversee the monitoring component of the treatment pathway. IGRA = interferon-gamma release assay; Hep Serology = hepatitis B and C serology. Source: Left hand figure reproduced with permission of former Public Health England (PHE), now UK Health Security Agency (UKSA).

The TBI pathway begins when an individual registers with a GP practice in Newham, where staff assess eligibility for TBI screening. Eligible migrants are offered an IGRA blood test to detect TBI. If the IGRA result is positive, a chest radiograph is arranged to exclude active TB disease. Once active disease is ruled out, patients undergo a clinical review in primary care, during which the GP explains the diagnosis and discusses potential benefits and side effects of treatment. The standard treatment offered in Newham is a 3-month course of 3HR, prescribed electronically by the GP. In the Newham model, accredited community pharmacies dispense medication and provide adherence support. Patients collect their medication monthly from one of several participating pharmacies, where trained pharmacists monitor progress, check for side effects, and report back to the GP. Throughout treatment, communication between GPs, pharmacists, and TB specialists ensures coordinated care and safety monitoring. Embedded within this model, an educational programme was developed, which involved periodic training for all health professionals in the borough, the production of novel patient education tools and electronic training material to support HCPs within primary care (Supplementary Data Figure). The TBI model has been formally evaluated within a cluster-randomised control trial (the CATAPuLT trial) which found no difference in TBI treatment completion between primary and secondary care. Importantly, the trial concluded that treatment in primary care is safe and reduces costs.^12^

The objective of this study was to evaluate the perceptions and experiences of health care professionals and stakeholders regarding the delivery of a community-based TBI programme. In addition, we aim to evaluate key barriers and enablers of the implementation of the programme.

## METHODS

Paper-based questionnaires were designed to evaluate health care professionals’ understanding of the TBI screening and treatment programme. Each questionnaire was divided into five main sections covering: 1) details specific to their role and training, 2) enablers and barriers to delivering a TBI programme in primary care, developed on the basis of expert opinion, 3) views around their role in the TBI programme, 4) assessment of TBI knowledge, and 5) feedback on the implementation of the TBI programme (Supplementary Data Figure). A combination of question modalities were used, including yes/no, multiple choice, Likert Scale (5- and 7-point ordinal scale to rate the degree to which responders agree or disagree with a specified statement), and free-text.^14^ Questionnaires were modified and tailored to the specific health care professional group. Each questionnaire included up to 40 questions and was anticipated to take 20 min to complete. Prior to utilisation, each questionnaire was piloted amongst representatives of each profession at one primary care clinic/general practice before being adapted and sent to the other settings. Questionnaires were circulated between August 2017 and February 2019, to health care professionals providing the service. This included general practitioners, community pharmacists, health care assistants, and practice nurses involved in the screening and treatment of TBI in Newham.

### Semi-structured interview

Semi-structured interviews were conducted between February 2019 and May 2019. Nine stakeholders from the Newham programme steering committee were invited to share their views of the programme. This included senior/lead pharmacists, GPs, TB nurses, and representatives from primary care and the CCG. Prospective participants were invited to interview either by email or telephone. Interviewees were selected based on their involvement in the delivery of the TBI screening programme to ensure that all aspects of the programme were covered, and where possible, data saturation achieved. An information leaflet was provided to all participants prior to the interview, including information about the study design, methodology, data analysis, data storage, and consent. Interviews with stakeholders lasted between 30 and 75 min utilising pre-determined open questions. Three were carried out by telephone, one via skype, and five were done face to face. Descriptive quantitative analysis was completed in Microsoft Excel.

### Ethical statement

Ethics approval was waived by the local ethics committee due to the study design and anonymity.

## RESULTS

Forty-three health care professionals completed the circulated questionnaire. This included 15 GPs, 15 health care assistants, 2 practice nurses, and 11 pharmacists. With regards to years of experience, eight GPs had been practising for more than 6 years and seven for less than 6 years; eight health care assistants and practice nurses for more than 6 years and nine for less than 6 years; and ten pharmacists for more than 6 years and one for less than 6 years.

**FIGURE 2.**
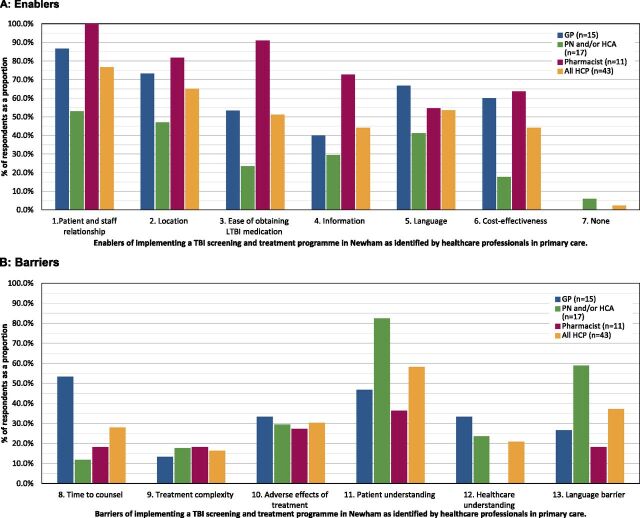
Key enablers and barriers of implementing a TB infection (TBI) screening and treatment programme in primary care. **A)** Enablers. Total sample size (n) = 43. Enablers 1. Relationship of patients with primary care staff; 2. Primary care locations are easier for patients to access than secondary care locations; 3. It is easier for patients to obtain their TBI medication; 4. Provision of information regarding TBI; 5. Reducing language barriers through the use of interpreters; 6. It is cheaper to deliver in primary care than secondary care; 7. There are no enablers. **B)** Barriers. Total sample size (n) = 43. Barriers identified: 8. Time required for counselling patients; 9. Complexity of TBI treatment; 10. Number of adverse effects of TBI treatment; 11. Patient understanding of TBI; 12. Health care professional understanding of TBI; 13. Language barriers between patient and health care professional. GP = general practitioner; PN = practice nurse; HCA = health care assistant.

Of the six key enablers included, the most commonly ranked by all HCP staff groups was having a perceived ‘good relationship’ between patients and primary care staff (n = 33, 76.7%) (Figure 2). This was followed by ease of access in terms of health care service location and medication. Individuals across all staff groups considered the use of interpreters to reduce language barriers as an enabler (n = 23, 53.5%). Barriers differed between staff groups; the most commonly listed barrier by GPs was time constraint on patient consultations (n = 8, 53.3%), whilst health care assistants and practice nurses (n = 14, 82.4%) and pharmacists (n = 4, 36.4%) more commonly reported limited patient understanding of TBI as a key barrier. Health care assistants and practice nurses regarded the explanation of the need for a diagnostic test as the most challenging aspect of the testing process (n = 8, 47.1%).

When asked on a five-point scale ‘TBI should be treated in primary care as opposed to secondary care’, all pharmacists (n = 11, 100%) strongly agreed and more than half of the GPs (n = 12, 67%) either agreed or strongly agreed. The majority of health care assistants and practice nurses were neutral (n = 9, 53%), and only six (35.3%) were in agreement. Despite this, almost all (n = 12, 67%) GP respondents agreed or strongly agreed with the statement that ‘GPs are pressurised to provide too many additional services in primary care’. This was higher than pharmacists who largely disagreed (n = 9, 81.8%) that ‘pharmacists are pressurised to provide too many additional services.’

Most health care professionals stated they received specific training for the Newham TBI screening and treatment programme (n = 38, 88.4%). The majority felt that this enabled them to carry out their role in the programme (n = 35, 87.5%). Pharmacists had most commonly undertaken TBI training through an online e-learning module (n = 7, 64%), whilst practice nurses and health care assistants were more likely to have had training by the CCG (n = 8, 47%) or attended specific nurse and health care assistant training sessions (n = 8, 47%). GPs were more likely to have had training provided by the CCG (n = 8, 53%). All staff groups felt more confident in treating patients after their respective training.

All pharmacists (n = 11, 100%) and the majority (n = 11, 73%) of GPs believed that pharmacists were best placed to check patient adherence to TBI medication. The majority (n = 9, 82%) of pharmacists believed that pill count was the best measure of adherence. Almost all (n = 10, 91%) pharmacists reported good adherence to TBI medication amongst their patients. Over half (n = 10, 67%) of the GPs felt that the comprehensive counselling provided by themselves in the initial consultation contributed to good adherence.

### Stakeholder views

All nine stakeholders invited participated in semi-structured interviews. Enablers and barriers to successful implementation of the community-based TBI programme were identified and divided into three levels: patient level, local health care facility level, and CCG level (Table 1). The importance of patient education of TB and TBI was emphasised as playing a key facilitating role in programme delivery, especially when available in multiple languages and thus addressing the needs of the population. Stakeholders also highlighted that public engagement events had the potential to improve screening uptake in the future. A ‘low level of patient knowledge of TB and TBI’ was identified by all stakeholders as a barrier to TBI screening and treatment uptake. Additional barriers identified were cultural stigma associated with TB and language barriers.

**TABLE 1.** Themes arising from key stakeholders on enablers and barriers of implementing a TB infection (TBI) screening and treatment programme.

Example	Patient level	Health care level	Clinical commissioning group (CCG) level
Enablers	TBI education by health care professionals	Training of health care professionals and administration staff	Effective communication between stakeholders
	TBI animation educational tool	Accessibility to specialist TB advice by TB specialist	Aggregate data collection and monitoring
Barriers	Low-level patient knowledge of TB or TBI	Lack of a shared electronic platform between GP and pharmacy	Uncertainty of rates of screening and treatment uptake
	Cultural stigma and language barriers in migrants	Inconsistent coding of date of entry to the UK	Communication between health care professionals

Interviews were conducted with stakeholders involved in the Newham TBI programme to evaluate views on enablers and challenges associated with its implementation and delivery.

At the local health care level, all stakeholders identified that TBI training sessions undertaken by health care professionals were a key enabling factor for implementing the programme. A recognised barrier was that not all migrants registered with GP practices had information of their date of entry to the UK on their medical record. At the time of the study, it was not mandatory to routinely collect and record this information, leading to inconsistent coding and thereby impeding the recognition of potentially eligible patients for TBI screening. Lack of access to a shared IT system between pharmacists and GPs was also considered a barrier to service implementation; stakeholders reported that pharmacists were unable to directly access blood results such as liver function tests and had to instead contact the GP practices by telephone to obtain them.

Factors identified as enabling the implementation of the TBI programme included effective communication between all stakeholders (GP practices, pharmacies, secondary care) and continual review of the TBI programme. Good clinical leadership was found to be of high importance to all, with many noting the opportunity to seek expert advice from GP and TB health care providers in secondary care as a major benefit to the TBI programme. Aggregate data collection and monitoring at a general practice level was considered another major enabler by all participants.

Stakeholders also noted difficulties with estimating TBI screening and treatment uptake for resource allocation to the programme. Overall, the majority of stakeholders interviewed were supportive of a primary care–based TBI screening and treatment programme being implemented in other high-TB-incidence areas nationally, though several stakeholders had concerns about whether the necessary infrastructure or services such as aggregate data monitoring would be available.

## DISCUSSION

This study, conducted in a London Borough implementing the UK’s national TB Screening and treatment programme, identified key barriers and enablers, from the perspective of health care professionals and stakeholders. We found that good rapport between health care professionals and patients, patient education, involvement of pharmacists, and access to specific skills and training were considered crucial enablers for the successful delivery of a TBI screening programme. Key challenges identified by both health care professionals and stakeholders were ‘low level of patient knowledge of TB and TBI’ and language barriers.

Lack of or reduced awareness about TBI and TBI screening programmes has previously been shown to lead to apprehension around screening and/or treatment as well as stigma within migrant communities.^10,15–18^ Language barriers together with low health literacy can lead to poor communication, ineffective consultations, and the inability to educate patients from certain demographics, thus preventing successful implementation of TBI screening and treatment.^19,20^ These barriers can be addressed by having longer appointment times, availability of interpreting services (face-to-face, telephone, or video-based), and the use of health advocates to improve health literacy and to provide cultural support.^16,21,22^ In addition, public awareness campaigns like the TBI animation film ‘Latent tuberculosis (TB): animated into action’ made specifically for the Newham project can increase TBI screening and treatment uptake. This health promotion tool aims to engage and inform new migrants from countries with high burden of TB about TBI and local NHS testing and treatment programmes. It is available in six languages.^23^ Patient education has been shown to lead to better TBI outcomes including adherence and completion of TBI treatment.^17,24–26^

Our semi-structured interviews highlight the importance of health care professional training and development in facilitating a successful TBI programme. This furthers a previous UK-based TBI screening survey that concluded despite GPs being supportive of TBI treatment in the community, only a small number felt confident to initiate or modify TBI treatment.^27^ In the Newham TBI screening and treatment programme, the majority of GPs surveyed felt that the specific training was sufficient to enable them to carry out their role with confidence. However, GPs surveyed perceived the TBI programme contributing to their high workload as they are expected to provide a significant number of specialist services, which is consistent with other studies highlighting the high work load of GPs.^28,29^

Previous studies have reported success in community pharmacy–based monitoring of adherence to new medications^30^ which is supported by our study. The majority of GPs and pharmacists felt that pharmacists were best placed to monitor adherence. Pharmacists also reported good adherence amongst their patients.

The strength of this study was that we were able to identify key barriers and enablers of a community-based TBI screening and treatment programme from a health care professional and stakeholder perspective. At the time of this study, and to our knowledge, this was the first process evaluation of a TBI screening and treatment programme conducted entirely in primary care. Although the Newham TBI programme demonstrated high screening uptake, treatment completion, and overall effectiveness, a key limitation was that the views and perceptions of patients were not systematically assessed as part of the programme evaluation.^13,31^ Despite this, available feedback indicated high levels of patient satisfaction, with participants reporting that care delivered by GPs and community pharmacies was acceptable and reassuring.

Our results suggest that in order to implement a primary care–based TBI screening and treatment model, it is essential to have the right service infrastructure and accurate coding to be able to provide aggregate data at practice level. Previous studies have also emphasised the need for continuous data collection and monitoring in screening programmes.^32^ We recommend that a TBI programme delivered in primary care should include regular education and awareness campaigns informing individuals at risk of TBI, particularly those from high-TB-incidence countries or communities with known barriers to accessing TBI screening and treatment. It is essential that regular training and refreshers for GPs, pharmacists, and reception/admin staff is provided. Embedding TBI screening into electronic health record prompts and facilitating TBI screening at patient GP registration is important. Finally, aggregate data monitoring including recording of performance metrics (screening uptake, treatment initiation, and completion) is vital to increase the effectiveness of a TBI programme (Table 2). Further research is needed to not only address barriers from an HCP and stakeholder perspective but also identify barriers from a service user perspective and to identify interventions to overcome these barriers to guarantee the success of a primary care–based TBI programme.

**TABLE 2.** Barriers and enablers of TB infection (TBI) screening and treatment programme in primary care.

Category	Example	Interventions
Patient-level barriers		
Knowledge and understanding of TBI	• Limited understanding of TBI in contrast to active TB	• Education and awareness campaigns informing patients about TB symptoms and why TBI screening matters
	• Limited knowledge of the risk of progression to active TB	• Face-to-face education by health care professionals
		• TBI information leaflets in multiple languages
		• Community awareness campaigns through migrant charities
Health care access	• Difficulty attending appointments due to lack of transportation, cost, or work commitments	• Opt-out TBI screening by health care professionals
	• Language barriers or low health literacy	
	• Lack of trust in health care systems	
Language barriers	• Lack of interpreters	• Availability of interpreters or health advocates from the same cultural background
		• TBI information material available in different languages
Cultural barriers	• Migrants eligible for screening may prioritise immediate social and economic needs such as employment, immigration, work, and housing and drop out of TBI services	• Integrate TBI screening/treatment programme with partner agencies (housing, social services, migrant support)
		• Delivery of culturally competent and linguistically appropriate care
Stigma and fear	• Misconceptions about TB infection	• Trusted community health care professionals or peer educators/ advocates to support patients
	• Stigma and fear of discrimination	• Holistic support by trained health care professionals
	• Poor adherence to preventive TB regimens	
Health care provider barriers		
Knowledge and training	• Limited familiarity with TBI screening guidelines	• Delivery of regular training and refreshers for all staff (GPs, pharmacists, reception/admin) about
		∘ TB eligibility
	• Uncertainty about management or follow-up after positive results.	∘ TBI pathway steps
		∘ Cultural competence
		∘ Confidence in TBI screening and treatment
		∘ Data recording
Perception of TBI screening		• Education on
		∘ Recognition of TBI screening as a key step in TB elimination
		∘ Identification of TB leads in GP practices promoting TB prevention in consultations
Time constraints	• GPs may face cultural and linguistic barriers, difficulties in identifying eligible patients	• Embedding TBI screening into electronic health record prompts
Health system barriers		
Infrastructure and resources	• Inadequate funding and infrastructure for screening and follow-up	• Embedding TBI screening at patient GP registration
		• Commissioning programmes for GPs and pharmacists to deliver the TBI programme
Lack of coordination between TBI stakeholders	• Poor communication between primary and secondary care	• Monitor performance metrics (screening uptake, treatment initiation/completion, pathway drop-offs) and use quality improvement methods to address barriers: regular TBI stakeholder meetings; TBI dashboards

## CONCLUSION

This study identifies health care professionals and stakeholders’ perceptions of barriers and enablers regarding the successful delivery of a TBI screening and treatment programme in primary care. Effective patient–provider relationships, targeted training for HCPs, and strong communication between multi-level stakeholders were perceived as key enablers, whilst limited patient knowledge and awareness of TB or TBI, time constraints for GPs, and communication challenges between HCPs were seen as barriers. These findings suggest that primary care–based TBI screening and treatment may be feasible when supported by trained staff, adequate resources, and collaborative stakeholder engagement experienced in TBI management. The insights from this study may help to inform targeted policy development aimed at strengthening the national TBI screening and treatment programme in the UK and similar settings globally.
